# Proximity Biotin Labeling Reveals Kaposi’s Sarcoma-Associated Herpesvirus Interferon Regulatory Factor Networks

**DOI:** 10.1128/JVI.02049-20

**Published:** 2021-04-12

**Authors:** Ashish Kumar, Michelle Salemi, Resham Bhullar, Sara Guevara-Plunkett, Yuanzhi Lyu, Kang-Hsin Wang, Chie Izumiya, Mel Campbell, Ken-ichi Nakajima, Yoshihiro Izumiya

**Affiliations:** a Department of Dermatology, School of Medicine, University of California Davis (UC Davis), Sacramento, California, USA; b Genome Center, Proteomics Core, Genome and Biomedical Sciences Facility, UC Davis, Davis, California, USA; c Department of Biochemistry and Molecular Medicine, School of Medicine, UC Davis, Sacramento, California, USA; d Viral Oncology and Pathogens-Associated Malignancies Initiative, UC Davis Comprehensive Cancer Center, Sacramento, California, USA; Lerner Research Institute, Cleveland Clinic

**Keywords:** KSHV, TurboID, proteomics, Kaposi’s sarcoma-associated herpesvirus, proteomics, proximity biotin ligation, SF3B1, human herpesviruses, interferons, vIRF

## Abstract

Studies on “hit-and-run” effects by viral proteins are difficult when using traditional affinity precipitation-based techniques under dynamic conditions, because only proteins interacting at a specific instance in time can be precipitated by affinity purification. Recent advances in proximity labeling (PL) have enabled identification of both static and dynamic protein-protein interactions. In this study, we applied a PL method by generating recombinant Kaposi’s sarcoma-associated herpesvirus (KSHV). KSHV, a gammaherpesvirus, uniquely encodes four interferon regulatory factors (IRF-1 to -4) that suppress host interferon responses, and we examined KSHV IRF-1 and IRF-4 neighbor proteins to identify cellular proteins involved in innate immune regulation. PL identified 213 and 70 proteins as neighboring proteins of viral IRF-1 (vIRF-1) and vIRF-4 during viral reactivation, and 47 proteins were shared between the two vIRFs; the list also includes three viral proteins, ORF17, thymidine kinase, and vIRF-4. Functional annotation of respective interacting proteins showed highly overlapping biological roles such as mRNA processing and transcriptional regulation by TP53. Innate immune regulation by these commonly interacting 44 cellular proteins was examined with small interfering RNAs (siRNAs), and the splicing factor 3B family proteins were found to be associated with interferon transcription and to act as suppressors of KSHV reactivation. We propose that recombinant mini-TurboID-KSHV is a powerful tool to probe key cellular proteins that play a role in KSHV replication and that selective splicing factors have a function in the regulation of innate immune responses.

**IMPORTANCE** Viral protein interaction with a host protein shows at least two sides: (i) taking host protein functions for its own benefit and (ii) disruption of existing host protein complex formation to inhibit undesirable host responses. Due to the use of affinity precipitation approaches, the majority of studies have focused on how the virus takes advantage of the newly formed protein interactions for its own replication. Proximity labeling (PL), however, can also highlight transient and negative effects—those interactions which lead to dissociation from the existing protein complex. Here, we highlight the power of PL in combination with recombinant KSHV to study viral host interactions.

## Abstract (precis)

Viral protein interaction with a host protein shows at least two sides: (i) taking host protein functions for its own benefit and (ii) disruption of existing host protein complex formation to inhibit undesirable host responses. Due to the use of affinity precipitation approaches, the majority of studies have focused on how the virus takes advantage of the newly formed protein interactions for its own replication.

## INTRODUCTION

Kaposi’s sarcoma herpesvirus (KSHV) infection is associated with endothelial Kaposi’s sarcoma (KS) (1, 2), B-cell malignancies such as primary effusion lymphoma (PEL), and AIDS-related multicentric Castleman’s disease (MCD) (3–6). In these cancer cells, KSHV mostly exhibits latent infection, in which the viral genes maintain silence to avoid recognition by the host immune system. However, some infected cells undergo spontaneous reactivation, in which KSHV produces infectious progeny virions. During lytic replication, KSHV produces infectious virions and facilitates transmission of the virus to neighboring cells; the process also increases the risk of the virus being recognized by the host immune system (7). Host innate immune system detect pathogens through binding of pathogen-associated molecular patterns (PAMPs) to pattern recognition receptors (PRRs). Several PRRs, such as IFI16 (8, 9), RIG-I (10–12), Toll-like receptor 9 (TLR9) (13), TLR3 (14), TLR4 (15), and NLRP1 (16), are known to detect KSHV-associated PAMPs. The recognition of KSHV DNA by PRRs subsequently leads to phosphorylation, dimerization, and nuclear translocation of interferon (IFN) regulatory factor 3 (IRF-3)/IRF-7. IRF-3/IRF-7 binds to DNA through its DNA binding domain (DBD), which results in secretion of cytokines and IFNs. In order to counteract the host response, KSHV encodes several immunomodulatory proteins, such as viral interferon regulatory factors (vIRFs) that inhibit the antiviral response and aid viral replication (17, 18).

KSHV genome encodes four vIRFs, vIRF-1 to -4. The N termini of vIRFs exhibit similarity to N termini of cellular IRFs; however, viral IRFs lack a key tryptophan residue, which is required for binding to DNA (19). The vIRF-1, vIRF-2, and vIRF-4 genes are inducible lytic genes, although vIRF-1 can also be found in a small portion of latently infected cells. In contrast, vIRF-3 (also known as LANA2) was discovered as a latent protein, and its expression remains unchanged during reactivation (20). Studies have shown that vIRFs counteract the host IFN response by interacting with cellular proteins. For example, vIRF-1 suppresses cellular IRF-3-mediated transcription by binding to p300, thereby preventing p300/CBP–IRF-3 complex formation (21, 22). vIRF-1 also promotes KSHV lytic replication by recruitment of USP7 (23). vIRF-2 was found to inhibit KSHV lytic gene expression by increasing the expression of cellular antiviral factors like promyelocytic leukemia nuclear bodies (PML) (24). Similarly, vIRF-3 suppresses KSHV reactivation by interacting with USP7, and the interaction also supports PEL cell growth (23). vIRF-4 also associates with IRF-7 and inhibits IRF-7 dimerization to suppress IFN production (25). These studies demonstrated different outcomes in different cell lines, suggesting the significance of implementing proteomic approaches that can reveal vIRF interaction networks more comprehensively.

Dynamic and stable protein-protein interactions are key to cellular processes and biological pathways. Affinity purification coupled with mass spectrometry (AP-MS) has been an invaluable method used to identify protein-protein interactions. However, AP-MS often fails to identify weakly or transiently interacting proteins. To overcome this drawback, enzyme-based proximity-based labeling (PL) approaches have been developed (26, 27). The approach provides sensitivity and specificity required to study dynamic protein-protein interaction. BirA_R118G_ (BirID) was the first proximity-based labeling enzyme identified in Escherichia coli which conjugates biotin to lysine residues of neighboring proteins (28). However, original BirID required the presence of biotin for several hours to be able to biotinylate a sufficient amount of proteins for analysis, thereby restricting its use for dynamic processes. Recently, two variants of BirID have been developed by directed evolution, named mini-TurboID (28 kDa) and TurboID (35 kDa), which allow proximity labeling in less than 10 min without significant toxicity (27). The TurboID-based approach has already been successfully employed in a wide variety of species, including mammalian cells (27, 29–32), *Drosophila* (33), plants (34–37), yeasts (38), and flies and worms (27).

In this study, we prepared recombinant 3×Flag-mini-TurboID-vIRF-1 and 3×Flag-mini-TurboID-vIRF-4 KSHV to biotinylate host and viral proteins in the vicinity of these two viral proteins. The proximity-labeling approach combined with mass spectrometry identified both previously identified cellular proteins and new host proteins as their possible interacting partners. Small interfering RNA (siRNA) screening of these interacting proteins showed that selective splicing factors function to suppress KSHV reactivation and are associated with antiviral responses.

## RESULTS

### Construction of 3×Flag-mini-TurboID-K9 and 3×Flag-mini-TurboID-K10 KSHV BAC16.

Biotin-labeled proximity labeling (PL) has emerged as a powerful method for probing various target proteins in a wide variety of species, including mammalian cells and unicellular organisms (27, 32, 33, 35, 37, 38). We thought that applying this technique to virology would be particularly beneficial, because viruses completely depend on host cell machinery for their replication. Indeed, many key cellular proteins, such as p53, were identified as virus-interacting proteins (39–41). Our major goal is thus to report utility of PL in conjunction with the recombinant KSHV bacterial artificial chromosome (BAC) system.

**FIG 1:**
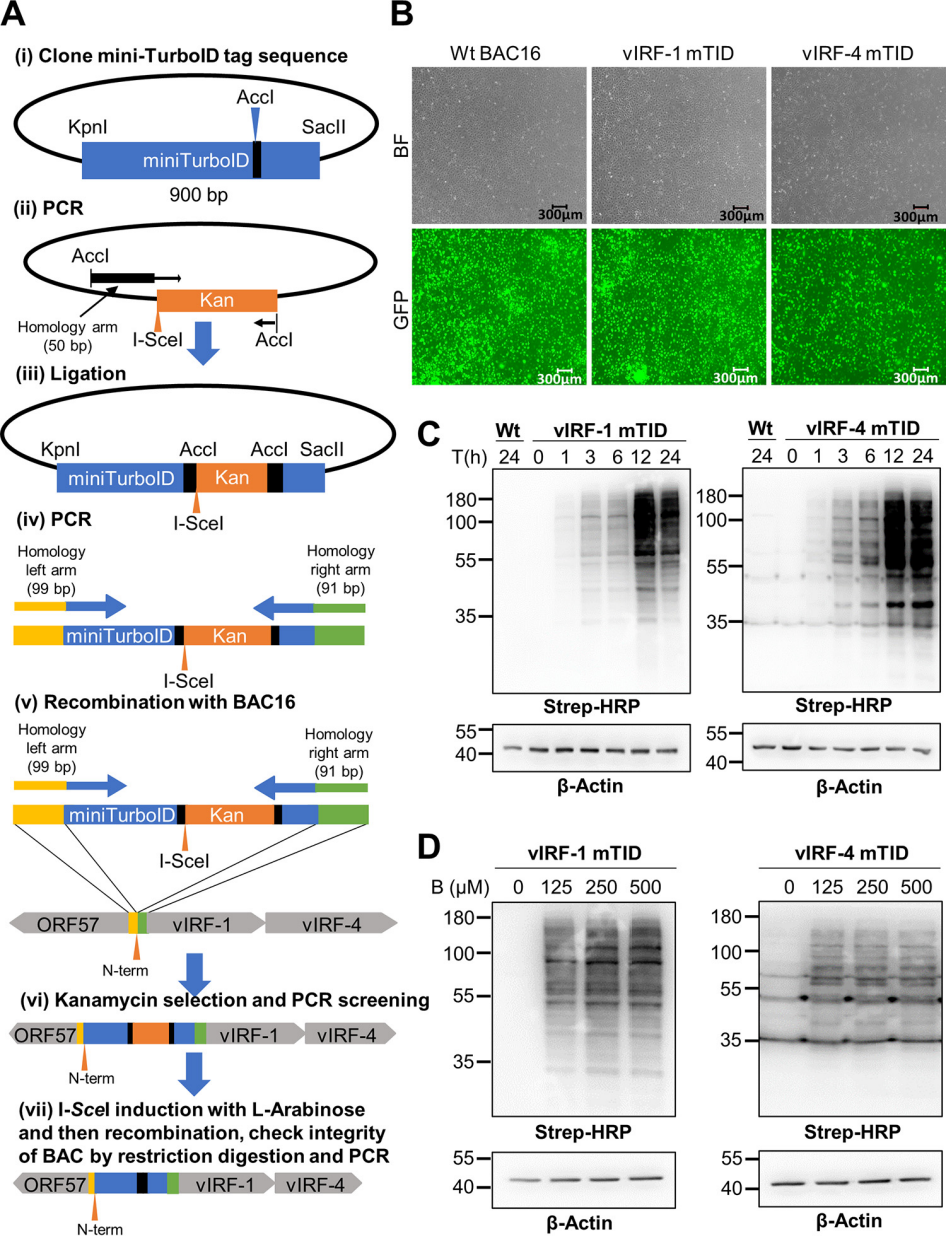
Engineering of mini-TurboID KSHVs. (A) Schematic diagram for construction of 3×Flag-mini-TurboID-K9 and 3×Flag-mini-TurboID-K10 with KSHV BAC16. (i) The codon-optimized cDNA fragment (900 bp) of mini-TurboID was synthesized and cloned into the pBS vector between the KpnI and SacII restriction enzyme sites. (ii) The kanamycin cassette with I-SceI recognition sequence along with 50 bp of homologous sequence was generated by PCR with pEP-Kan plasmid as a template and cloned into the AccI restriction enzyme site. (iii to v) The resulting plasmid was fully sequenced and used as a template to generate a DNA fragment for homologous recombination with BAC16 inside bacteria. (vi and vii) After confirmation of insertion at correct site by colony PCR screening, the kanamycin cassette was deleted by recombination with induction of I-SceI in bacteria by incubation with l-arabinose. Correct insertion of the mini-TurboID and integrity of BAC DNA were confirmed by sequencing of PCR-amplified fragments and restriction digestions. Primers and the DNA fragment used are listed in Table 1. (B) Generation of Wt BAC16, vIRF-1 mTID, and vIRF-4 mTID stable cells. iSLK cells were transfected with Wt BAC16, 3×Flag-mini-TurboID-K9, and 3×Flag-mini-TurboID-K10 KSHV BAC16 and stably selected with hygromycin (1 mg/ml). Green fluorescent protein (GFP) images show iSLK cells latently infected with Wt BAC16, 3×Flag-mini-TurboID-K9, and 3×Flag-mini-TurboID-K10 KSHV BAC16. BF, bright field. (C) Protein biotinylation with timescale. Wt BAC16 (Wt), vIRF-1 mini-TurboID (vIRF-1 mTID), and vIRF-4 mini-TurboID (vIRF-4 mTID) cells were stimulated with Dox (1 µg/ml) and NaB (3 mM) for 24 h, followed by incubation with d-biotin (500 µM) for the indicated periods. Activity of mini-TurboID was analyzed by immunoblotting using streptavidin (Strep)-HRP conjugate. β-Actin was used as the input control. T(h), time (hours). (D) Determination of the optimal amount of exogenous d-biotin (B) for labeling. vIRF-1 mTID and v-IRF-4 mTID cells were stimulated with Dox (1 µg/ml) and NaB (3 mM) for 24 h, followed by incubation with the indicated concentration of d-biotin for 1 h. Activity of mini-TurboID was examined by immunoblotting using streptavidin-HRP conjugate. β-Actin was used as the input control.

To generate recombinant KSHV conveniently, we first prepared a template plasmid, which is used to create PCR fragments for recombination. The template encodes a 3×Flag tag at the N terminus of mini-TurboID and kanamycin cassettes in mini-TurboID coding region as an excisable format with I-SceI induction. The 3×Flag-mini-TurboID-kana DNA fragment was amplified with primers with homology arms, and amplified fragments were then used for recombination by using a two-step recombination approach as previously described (42) (Fig. 1A). The wild-type (Wt) BAC16, 3×Flag-mini-TurboID-K9, and 3×Flag-mini-TurboID-K10 BAC16 were directly transfected into iSLK cells and selected with hygromycin (1 mg/ml) to generate iSLK cells harboring latent Wt BAC16 KSHV (named Wt BAC16 cells), 3×Flag-mini-TurboID-K9 KSHV (named vIRF-1 mTID cells), and 3×Flag-mini-TurboID-K10 KSHV (named vIRF-4 mTID cells), respectively (Fig. 1B).

We first examined optimal duration of incubation time, and the amount of exogenous biotin for efficient labeling was determined. To test biotin ligase activity, Wt BAC16, vIRF-1 mTID, and vIRF-4 mTID cells were reactivated with doxycycline (Dox) and sodium butyrate (NaB) for 24 h. Subsequently, stable cells were incubated with 500 µM biotin for various periods, and Wt BAC16 cells were used as a negative control. Labeling was terminated by incubating cells at 4°C and removing excess biotin. Immunoblot analysis using horseradish peroxidase (HRP)-conjugated streptavidin showed multiple biotinylated proteins, indicating successful labeling with vIRF-1- and vIRF-4-tagged mini-TurboID. Mini-TurboID biotinylated proteins within 1 h after addition of exogenous biotin, and signal intensity gradually increased along with incubation time. Wt BAC16 cells did not show biotinylation signal even after 24 h of biotin incubation, indicating that the signal is specific to mini-TurboID (Fig. 1C, lanes 1). Next, we optimized the exogenous biotin concentration. The vIRF-1 and vIRF-4 mTID cells were reactivated for 24 h, incubated with different amounts of biotin (0, 125, 250, and 500 µM) for 1 h, and subsequently monitored for their biotinylation signal in whole-cell lysates using streptavidin immunoblotting. Untreated cells in the absence of biotin were used as a negative control. Comparable levels of signal intensity were observed from 125 µM to 500 µM, suggesting that saturation of protein biotinylation occurs at 125 µM (Fig. 1D). Considering that only a small proportion of cells were reactivating in a dish, we concluded that there was a sufficient amount of biotinylation in a reactivating cell for protein identification. For the following studies, we decided to use a saturating amount of biotin (500 µM) for 1 h of incubation.

### Recombinant vIRF-1 mTID and vIRF-4 mTID KSHV gene expression.

**FIG 2:**
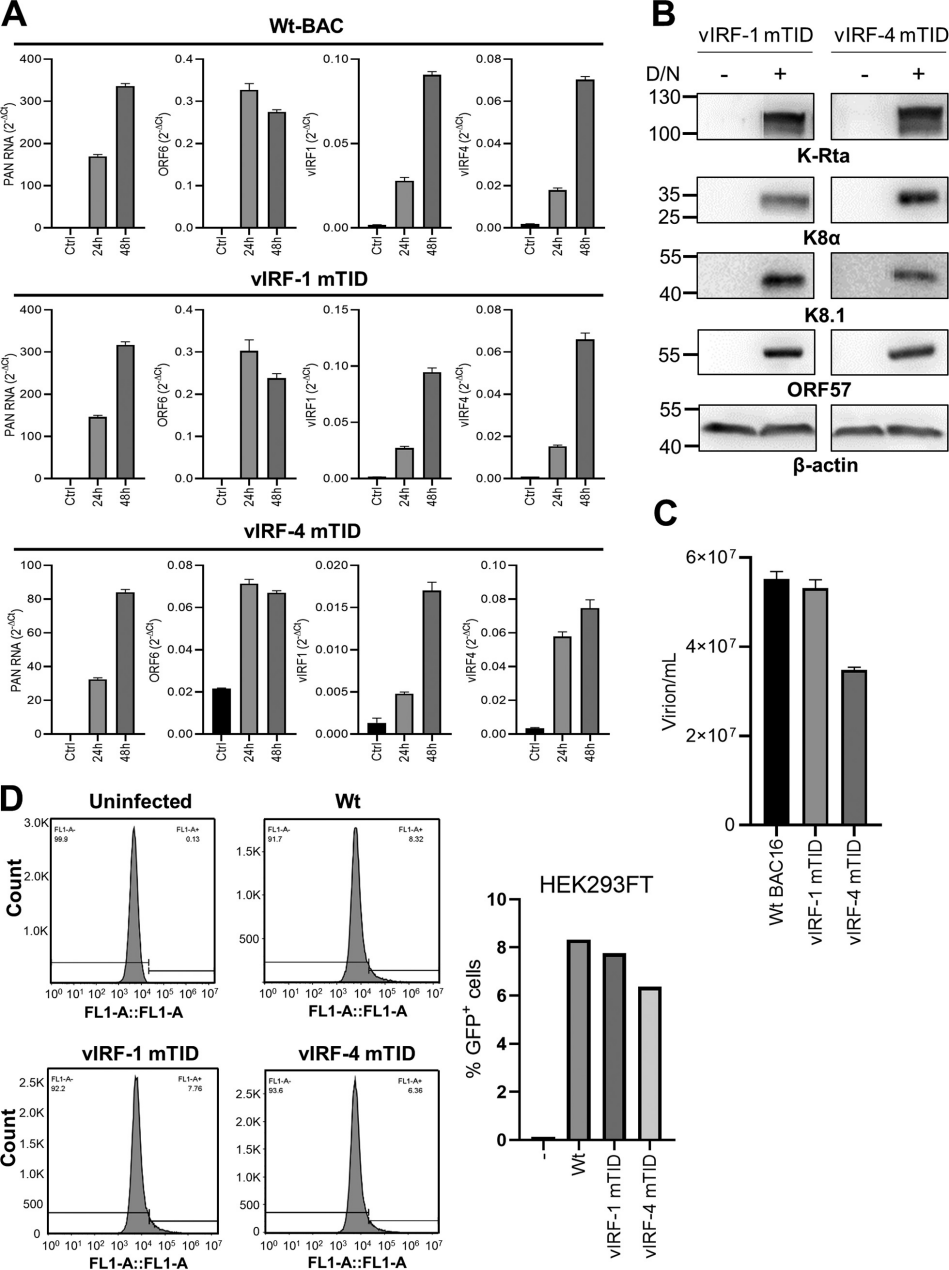
Viral gene expression and infectious virion production. (A) Viral gene expression for vIRF-1 and vIRF-4 mTID cells. Wt BAC16, vIRF-1 mTID, and vIRF-4 mTID cells were stimulated with Dox (1 µg/ml) and NaB (3 mM) for 24 h and 48 h. Total RNA was purified and subjected to real-time PCR for the indicated genes. Gene expression is shown as a threshold cycle (2^−ΔCT^). 18S rRNA was used as an internal standard for normalization. (B) Viral protein expression in vIRF-1 and vIRF-4 mTID cells. vIRF-1 mTID and vIRF-4 mTID cells were stimulated with Dox (1 µg/ml) and NaB (3 mM) for 24 h. Total cell lysates were subjected to immunoblotting using KSHV proteins and β-actin protein-specific antibodies. (C) Viral particle production. Wt BAC16, vIRF-1 mTID, and vIRF-4 mTID cells were stimulated with Dox (1 µg/ml) and NaB (3 mM) for 96 h. Encapsidated viral DNA copy number in the supernatant was quantified by quantitative PCR. (D) *De novo* infection. HEK293FT cells were infected with Wt, vIRF-1, and vIRF-4 mTID virus at 1 genomic copy of virus per cell. Flow cytometry was performed to quantify the number of GFP-positive cells. The bar graph shows the percentage of GFP-positive cells.

We next verified the induction of viral genes to ensure that tagging K9 or K10 genes with 3×Flag-mini-TurboID has little effect on viral gene expression or virion production. We compared induction of viral inducible genes in vIRF-1 and vIRF-4 mTID cells with that in Wt BAC16 cells. We stimulated Wt BAC16, vIRF-1, and vIRF-4 mTID cells with Dox and NaB and performed quantitative PCR (qPCR) for selected KSHV genes. We observed induction of KSHV lytic genes, PAN RNA, ORF6, vIRF-1, and vIRF-4 in all three cell lines. The viral gene expression in vIRF-1 mTID cells was comparable to that in Wt BAC16 cells, while v-IRF-4 mTID showed approximately 4 to 5 times lower levels of gene expression (Fig. 2A). In addition, we verified induction of selected lytic KSHV proteins at 24 h (Fig. 2B) and confirmed the production of virion particles in culture supernatants. We reactivated the Wt BAC16, vIRF-1, and v-IRF-4 mTID cells for 96 h and quantified the virion particles in the culture supernatant. The results showed comparable levels of virion particles in Wt BAC16 cells and vIRF-1 mTID cells, whereas vIRF-4 mTID cells showed approximately 2-fold-lower virion production (Fig. 2C). Finally, viral genomic copy numbers were adjusted to one viral genome copy per cell and HEK293FT cells were infected to examine infectivity by flow cytometry. The results demonstrated similar levels of infectivity with all three viruses (Fig. 2D). These results suggested that both vIRF-1 and vIRF-4 mTID viruses were able to complete the viral lytic replication cycle, although the mini-TurboID tag interferes with viral gene expression in vIRF-4 mTID virus.

### Proximity biotin labeling with vIRF-1 and vIRF-4.

**FIG 3:**
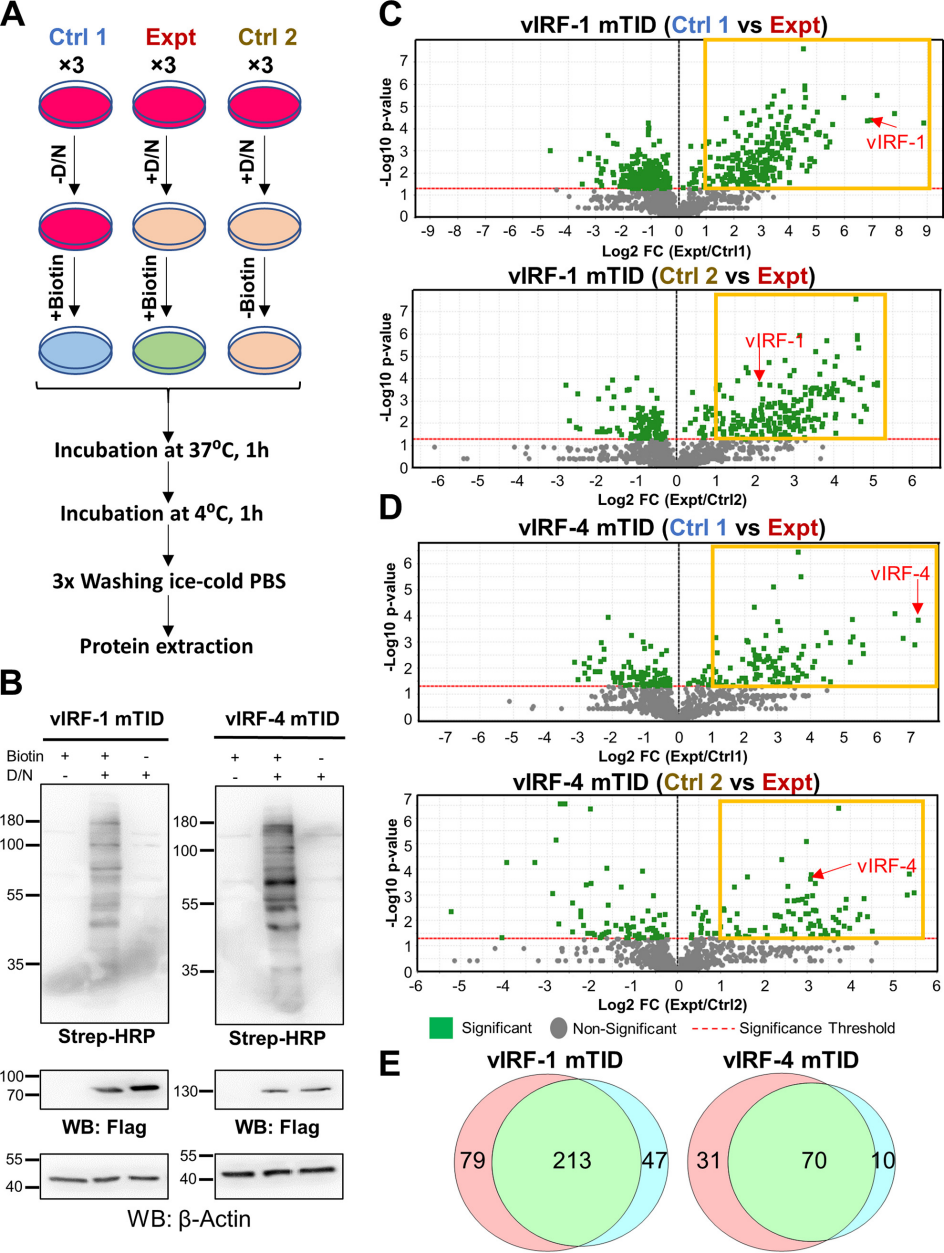
Proteins in close proximity to vIRF-1 and vIRF-4. (A) Schematic workflow for experimental setup. Three biological replicates for each sample were analyzed by LC-MS/MS analysis. The plus and minus signs indicate presence and absence, respectively. Ctrl, control; Expt, experimental; D/N, Dox (1 µg/ml) and NaB (3 mM). (B) Confirmation of biotinylation. The cell lysate from one of the three biological replicates was subjected to immunoblotting using streptavidin-HRP conjugates, Flag antibody, and β-actin antibody. (C and D) Identification of proteins in close proximity to vIRF-1 and vIRF-4. Volcano plots show differential proteins profiles in Ctrl 1 and Expt samples and in Ctrl 2 and Expt samples for vIRF-1 (C) and vIRF-4 mTID cells (D). Identified and quantified biotinylated peptides are plotted as log_2_ fold change (Expt/Ctrl 1) or (Expt/Ctrl 2) versus −log_10_ *P* value. Biotinylated peptides for vIRF-1 (C) and vIRF-4 (D) are shown with red arrows. Yellow boxes indicate selected peptides with a fold change of >2 and a *P* value of <0.05. (E) Venn diagram comparing proteomic lists between Ctrl 1 versus Expt samples and Ctrl 2 versus Expt samples.

For proximity protein labeling, three replicate samples were prepared for both vIRF-1 and vIRF-4 mTID cells. Cells were reactivated with Dox and NaB for 24 h, followed by addition of biotin for 1 h. Two sets of controls were also processed concurrently to rule out nonspecific precipitations. In the first set, the cells were left without triggering reactivation, followed by incubation with biotin (+Biotin) to rule out nonspecific protein binding with biotin (Ctrl 1). For the second set, cells were reactivated with Dox and NaB for 24 h and incubated for additional 1 h in the absence of biotin (−Biotin) to rule out nonspecific interaction with streptavidin beads (Ctrl 2). A schematic workflow for the experiment is presented in Fig. 3A. We confirmed the biotinylation signal by streptavidin blotting and vIRF-1 and vIRF-4 expression by using anti-Flag antibody (Fig. 3B). The streptavidin blotting showed differences in biotinylation signal between vIRF-1 and vIRF-4; this is likely due to amount of vIRF-1 and vIRF-4 protein in the cell and/or amount of neighboring cellular proteins and their stabilities. Whole-cell lysates from vIRF-1 and vIRF-4 mTID cells were further used for enrichment of biotinylated protein using magnetic streptavidin beads. The enriched proteins were eluted from the streptavidin beads using trypsin on-bead digestion overnight. The biotinylated peptides in the experimental (Expt) samples were compared with those in Ctrl 1 and Ctrl 2 independently to remove background noise. We designated proteins with *P* values of <0.05 and fold change (FC) of >2 over both Ctrl1 and Ctrl2 as positive hits (Fig. 3C). Based on our criterion setting, we identified 213 and 70 as possible interacting proteins with vIRF-1 and vIRF-4 mTID cells, respectively (see Tables S1 and S2 in the supplemental material).

### vIRF-1 and vIRF-4 target pathway analysis.

**FIG 4:**
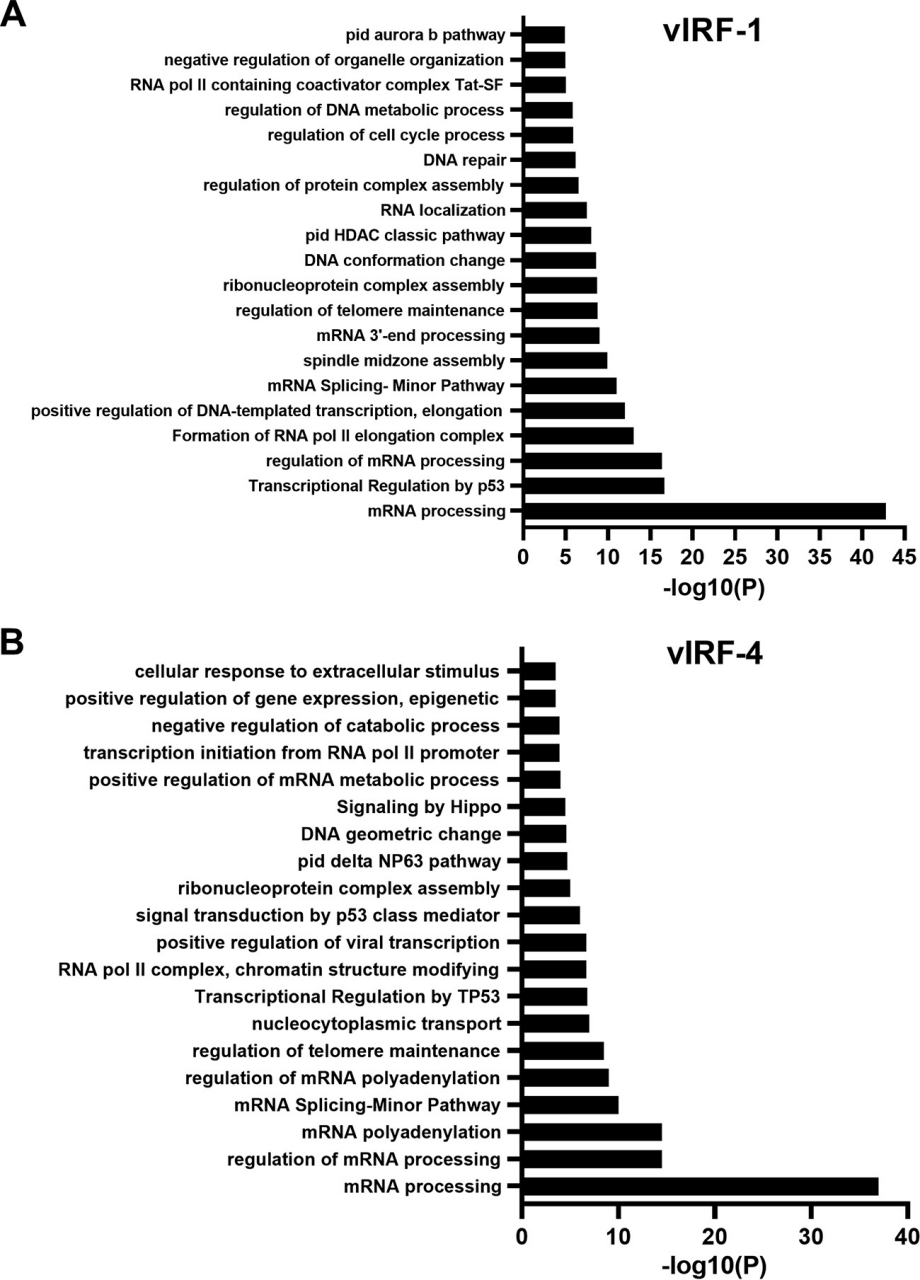
Pathway analysis for vIRF-1- and vIRF-4-interacting proteins. Shown are the top nonredundant enrichment clusters for vIRF-1-interacting (A) and vIRF-4-interacting (B) proteins using a Metascape bar graph (61).

Next, gene ontology (GO) analysis was performed for proteins possibly interacting with vIRF-1 and vIRF-4. The vIRF-1 interactome revealed significant enrichment for functions related to mRNA processing, transcription regulation by TP53, regulation of mRNA processing, and formation of RNA polymerase II (Pol II) elongation complex. The top 20 enriched GO terms are presented in Fig. 4A. Similarly, GO analysis for the vIRF-4 revealed again enrichment of mRNA processing, regulation of mRNA processing, mRNA polyadenylation, and mRNA splicing (Fig. 4B). Consistent with the fact that vIRF-1 and vIRF-4 have overlapping biological functions, we indeed found highly overlapping pathways that are associated with vIRF-1- and vIRF-4 precipitated proteins.

### Regulation of KSHV reactivation by commonly targeted cellular proteins.

**FIG 5:**
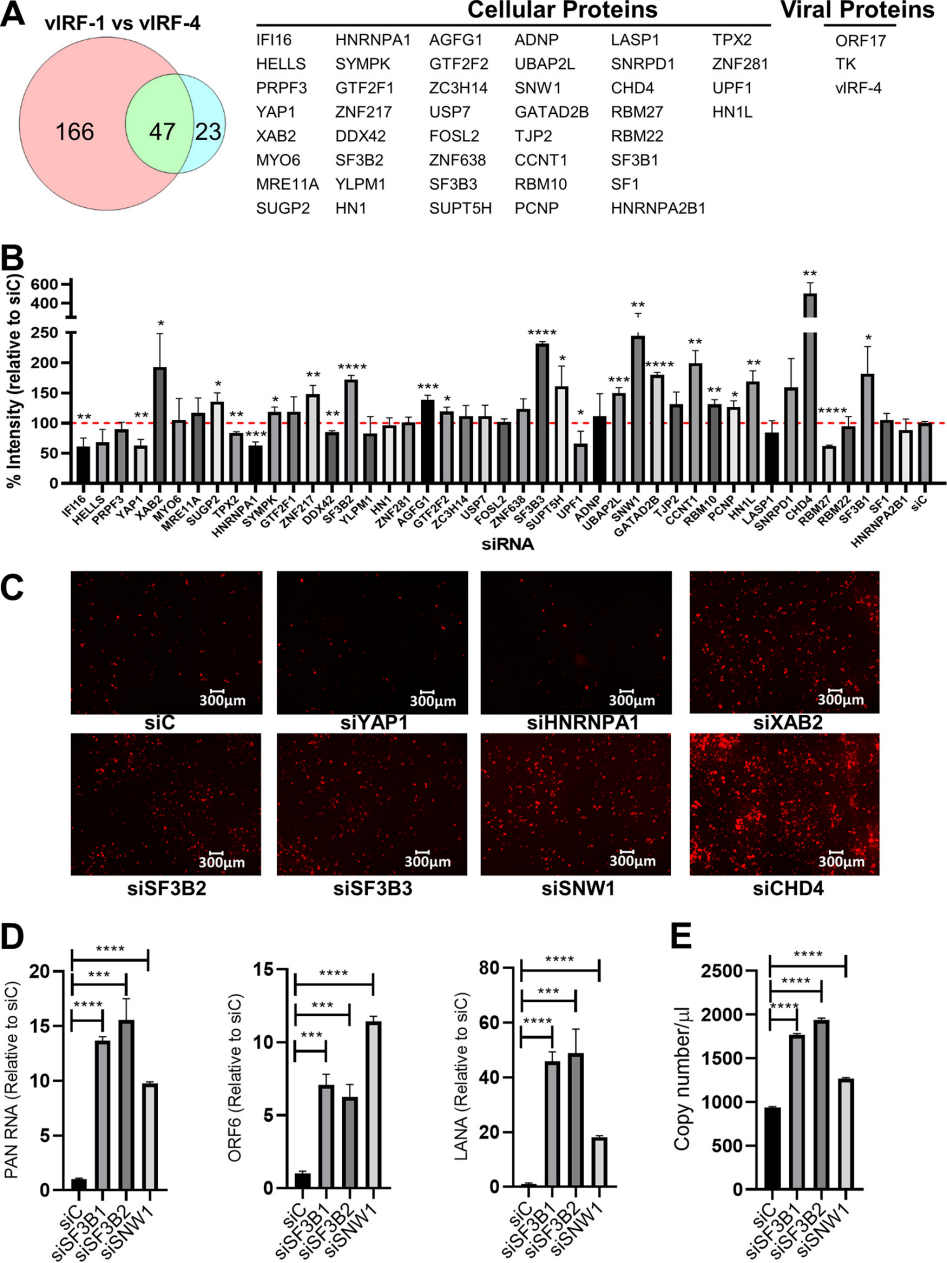
Splicing factor 3B (SF3B) subunits are suppressors of KSHV reactivation. (A) Common proteins between vIRF-1 and vIRF-4. The Venn diagram depicts the number of proteins that are identified with proximity protein labeling. Lists of 44 cellular proteins and 3 viral proteins interacting with both vIRFs are also shown. (B) Genes regulating KSHV reactivation. Five picomoles of individual siRNAs was transfected into iSLK.r219 cells for 48 h, followed by reactivation with Dox (1 µg/ml) and NaB (3 mM) for 24 h. Relative RFP signal intensity over control siRNA (siC) is shown. Red line represents 100% intensity signal. *, *P* ≤ 0.05; **, *P* ≤ 0.01; ***, *P* ≤ 0.001; ****, *P* ≤ 0.0001. (C) Microscopy imaging. Representative RFP images for panel B are shown. (D) Quantification of viral gene expression. Five picomoles each of siC, siSF3B1, siSF3B2, and siSNW1 was transfected into iSLK.r219 cells for 48 h, followed by reactivation with Dox (1 µg/ml) and NaB (3 mM) for 24 h. PAN RNA, ORF6, and LANA gene expression was quantified using real-time PCR. ***, *P* ≤ 0.001; ****, *P* ≤ 0.0001. (E) Quantification of progeny virus. Five picomoles each of siC, siSF3B1, siSF3B2, and siSNW1 was transfected into iSLK.r219 cells for 48 h, followed by reactivation with Dox (1 µg/ml) and NaB (3 mM) for 96 h. Viral copy number was quantified from tissue culture supernatants using real-time PCR. ***, *P* ≤ 0.001; ****, *P* ≤ 0.0001.

Previous studies demonstrated that both vIRF-1 and vIRF-4 regulate interferon pathways (18, 20). We thus hypothesized that commonly targeted cellular proteins by the two viral IRFs may play an important role in interferon responses. The results showed that 123 and 23 proteins interacted exclusively with vIRF-1 and vIRF-4, respectively, and 47 proteins were found to interact with both vIRF-1 and vIRF-4. Of these 47 proteins, 44 were cellular proteins, whereas 3 were viral proteins (Fig. 5A). The iSLK.219 cell line was employed to examine the role of these cellular proteins in KSHV replication. iSLK.219 cells carry a recombinant rKSHV.219 virus encoding a constitutively expressed green fluorescent protein (GFP) and a PAN RNA promoter-driven red fluorescent protein (RFP) reporter in the viral genome, allowing us to monitor lytic promoter activation. We used siRNA to knock down the 44 cellular genes, followed by KSHV reactivation by treatment with Dox and NaB to induce K-Rta expression. We found that knockdown of 17 genes enhanced KSHV PAN RNA promoter activation, while knockdown of 6 genes decreased the activation (Fig. 5B). The corresponding RFP images of selected knockdown experiments are shown in Fig. 5C, and the results were further confirmed by quantifying the viral mRNAs after knockdown of selected genes, i.e., the splicing factor 3B1 (SF3B1), SF3B2, and SNW1 genes (Fig. 5D). Consistent with increased viral gene expression, the viral DNA copy number in the culture supernatant was increased by knockdown of the SF3B1, SF3B2, or SNW1 gene (Fig. 5E). Taken together, our results suggest that certain splicing factors have a role in restricting KSHV gene expression during reactivation, in addition to their biological roles in general host gene transcription.

### SF3B subunits are important for IFN gene expression.

**FIG 6:**
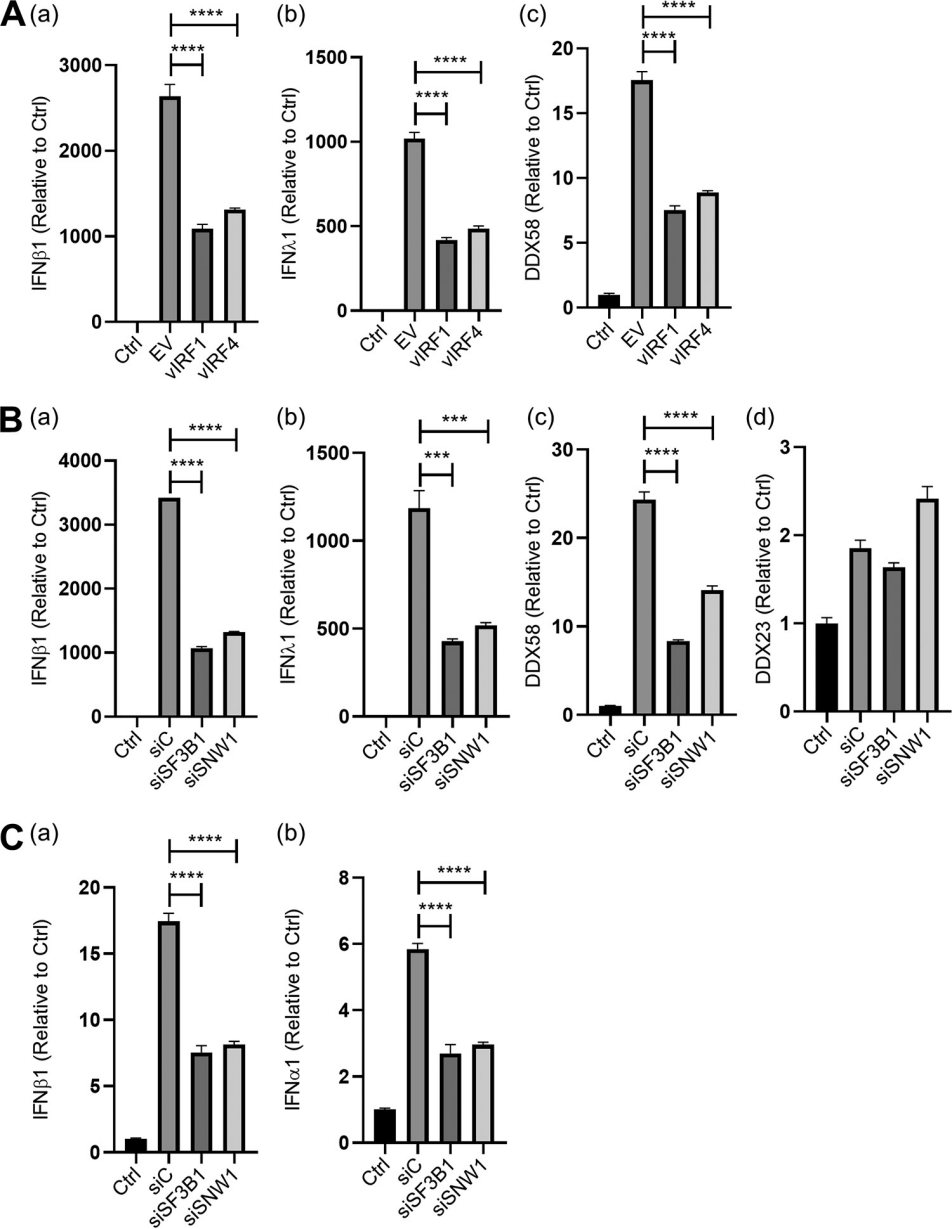
SF3B1 and SNW1 are suppressors of IFN-β1 transcription. (A) vIRF1 and vIRF4 expression plasmids were transfected into HEK293FT cells for 48 h, followed by poly(I·C) transfection. Twenty-four hours after poly(I·C) transfection, total RNA was harvested and IFN-related IFN-β1 (a), IFN-λ1 (b), and DDX58 (c) mRNAs were measured. (B) Five picomoles of siC, siSF3B1, or siSNW1 was transfected into HEK293FT cells for 48 h, followed by poly(I·C) transfection. Twenty-four hours after poly(I·C) transfection, total RNA was harvested and IFN-related IFN-β1 (a), IFN-λ1 (b), and DDX58 (c) mRNAs or nonrelated DDX23 mRNA (d) was measured. (C) Five picomoles of siC, siSF3B1, or siSNW1 was transfected into iSLK.r219 cells for 48 h, followed by KSHV reactivation by Dox (1 µg/ml) and NaB (3 mM). Forty-eight hours postreactivation, total RNA was harvested and IFN-related IFN-β1 (a) and IFN-α1 (b) mRNAs were measured. ***, *P* ≤ 0.001; ****, *P* ≤ 0.0001. siC, nontargeting siRNA.

Previous reports showed that the KSHV gene transcripts are sensed by RIG-I-like receptors (11). Poly(I·C) is a synthetic double-stranded RNA (dsRNA) polymer which is recognized by RIG-I, leading to strong induction of interferons and interferon-stimulated genes (ISGs). Because KSHV vIRFs are known to counteract IFN responses, we first examined vIRF-1 and vIRF-4 functions in interferon responses that are triggered by poly(I·C). vIRF-1 and vIRF-4 overexpression in HEK293FT cells resulted in inhibition of IFN-β1, IFN-λ1, and DDX58 in response to poly(I·C) transfection (Fig. 6Aa to Ac). Next, we examined the role of SF3B1 and SNW1 in regulation of the same IFN response. For this, SF3B1 and SNW1 were knocked down in HEK293FT cells, followed by poly(I·C) transfection. Consistent with our hypothesis that vIRFs interact with SF3B1 and SNW1 to inhibit IFN pathways, knockdown of SF3B1 and SNW1 also inhibited expression of type I interferon (IFN-β1) and type III interferon (IFN-λ1), as well as an interferon downstream target (DDX58) (Fig. 6Ba to Bc) but not the non-IFN target gene (Fig. 6Bd). Although we cannot rule out whether the IFN inhibition is mediated by the SF3B1 or SNW1 interactions, the results still suggested that both SF3B1 and SNW1 are involved in IFN pathway regulation. Knockdown of SF3B1 or SNW1 also led inhibition of type I interferons IFN-β1 and IFN-α1 during KSHV reactivation in iSLK.r219 cells (Fig. 6C). Together, these results may suggest that vIRF-1 and vIRF-4 interact with SF3B1 and SNW1 to modulate host antiviral immune responses.

## DISCUSSION

We applied a mini-TurboID-based system for studying the virus and host protein interaction. By constructing mini-TurboID as an integral component of the KSHV BAC16 recombination system, we demonstrated the utility of a new approach to identify protein interaction networks. We believe that this approach improves the reproducibility of identifying interacting proteins, because tight interaction between biotin and streptavidin allows us to wash magnetic beads under very stringent conditions and remove nonspecific or indirect protein interactions. The high reproducibility can be seen in our biological triplicate samples (Fig. S1).

To conveniently generate mini-TurboID-tagged viruses, we first generated template plasmids, similar to the strategy used for Rainbow-KSHV (43). With a plasmid template, homology arms can be added to primer pairs and the resultant PCR product is used for recombination (Fig. 1A). The targeted parental BAC16 genome can also be wild-type or mutant BAC16, and/or Rainbow-KSHV, which allows us to examine the formation of protein complexes during viral replication stages and also the effects of specific mutations on the protein interactions. In this study, however, we used vIRF-1 and vIRF-4 as bait for validating the efficiency of PL. vIRF-1 and vIRF-4 were selected because of their known role in regulation of the innate immune response during KSHV reactivation, and multiple interacting proteins that can be used as confirmation have been identified (18, 23, 25, 44). Consistent with previous studies, vIRF-1 and vIRF-4 were found to be physically neighboring cellular proteins that function in p53 transcriptional regulation (45). A study showed that vIRF-1 interacts with p53 to inhibit its transcriptional activation (44). Although our studies did not include precipitation of p53, we identified p53BP1 (p53 binding protein 1) as a possible partner of vIRF-1. We found USP7 in both vIRF-1 and vIRF-4 samples, validating the PL approach (23). In addition to the reported cellular proteins, we also confirmed SF3B1-vIRF interactions in transiently transfected 293FT cells (Fig. S2). The studies showed that transiently expressed Flag-tagged vIRFs were coprecipitated with endogenous SF3B1 protein.

After learning that the mini-TurboID approach efficiently biotinylated cellular proteins, we tagged various other KSHV genes with mini-TurboID using the same approach. However, we found that the efficacy of biotin labeling varies significantly among different viral proteins. For example, mini-TurboID-ORF57 robustly increased biotinylated protein in total lysates with as little as 1 h of d-biotin incubation, while biotinylation by mini-TurboID-ORF50 was barely detectable in the same time frame. For this study, we also generated and tested vIRF-2 and vIRF-3 mini-TurboID constructs; however, the level of biotinylation was much lower using the same amount of d-biotin and incubation periods, leading us to drop these genes from our analyses. Differences in efficacy of biotinylation have also been observed in prior studies (27), and abundance of viral protein expression during reactivation and subcellular nuclear localization seemed to have strong effects on the outcome of biotinylation.

Our PL studies showed that a large portion of interacting host proteins (36%) were related to mRNA processing. Of these RNA processing proteins, SF3B1, SF3B2, and SF3B3, components of the SF3B complex, clearly stood out in our siRNA screening. The SF3B complex is a component of the functional U2 small nuclear ribonucleoprotein (snRNP), which recognizes the exon/intron junctions and facilitates spliceosome assembly (46). Even though the SF3B1 gene is one of many cellular genes involved in RNA splicing, it has been specifically identified as a commonly mutated gene in myelodysplastic syndrome (MDS), at frequencies of 25 to 30% (47–49). Recent studies also showed that SF3B1 mutations increase R-loop formation and DNA damage (50). In this study, we found that SF3B1 knockdown inhibited IFN gene expression 3- to 4-fold and also enhanced KSHV reactivation. Indeed, SF3A1 and SF3B1 were previously reported to play a role in the innate immune response to TLR ligands. The study showed that SF3A1 and SF3B1 are necessary to increase production of interleukin 6 (IL-6) and IFN-β by modulating the splicing of MyD88, an important adaptor molecule for the TLR signaling pathway (51). It remains unknown how SF3B1 family regulates selected spliced genes. Based on the reported study and ours, we think that targeting the splicing complex might be a previously uncharacterized mechanism for KSHV to modulate host immune responses. Further studies on regulation of SF3B complex formation during KSHV reactivation and/or IFN stimulation with PL will clarify underlying mechanisms of SF3B family proteins in KSHV replication and IFN regulation.

In addition to SF3 complex, several other mRNA processing factors, like XAB2, SNRPD1, SNW1, RBM10, SYMPK, and GTF2F2, were found to suppress KSHV reactivation (Fig. 5). A recent study showed that SNW1 interacts with IKKγ, the regulatory subunit of the IκB kinase (IKK) complex. SNW1 increases production of IL-6, IFN-β, and MX1 by enhanced activation of NF-κB and phosphorylation of TBK1 in response to influenza A virus and poly(I·C) (52). Influenza A virus and poly(I·C) are recognized by the innate immune sensor RIG-I, and RIG-I plays an important role in suppressing KSHV reactivation by sensing KSHV DNA (11, 12, 53, 54). We found that knockdown of SNW1 indeed enhanced KSHV replication (Fig. 5D), and we think that this effect could be due to downregulation of IFN-β (Fig. 6).

In summary, using mini-TurboID KSHV with vIRFs as bait, we could successfully probe cellular proteins that play a role in innate immune responses. We propose that combination of mini-TurboID with the recombinant KSHV BAC system is a powerful tool to identify cellular proteins that play an important role in KSHV replication.

## MATERIALS AND METHODS

### Chemicals.

Dulbecco’s modified minimal essential medium (DMEM), fetal bovine serum (FBS), phosphate-buffered saline (PBS), trypsin-EDTA solution, 100× penicillin–streptomycin–l-glutamine solution, and streptavidin-HRP conjugate were purchased from Thermo Fisher (Waltham, MA). Puromycin and G418 solution were obtained from InvivoGen (San Diego, CA). Hygromycin B solution was purchased from Enzo Life Science (Farmingdale, NY). Anti-ORF57, anti-K8, and anti-K8.1 antibodies were purchased from Santa Cruz Biotechnology Inc. (Santa Cruz, CA). Anti-K-Rta antibody was described previously (55). All other chemicals were purchased from Millipore-Sigma (St. Louis, MO) unless otherwise stated.

### Cells, siRNA transfection, and reagents.

iSLK.219 cells were maintained in DMEM supplemented with 10% FBS, 10 μg/ml of puromycin, 400 μg/ml of hygromycin B, and 250 μg/ml of G418. iSLK cells were maintained in DMEM supplemented with 10% FBS, 1% penicillin-streptomycin solution, and 10 μg/ml of puromycin. iSLK cells were obtained from Don Ganem (Novartis Institutes for Biomedical Research). HEK293FT cells were grown in DMEM containing 10% FBS and 1% penicillin-streptomycin. Transfection of siRNA in iSLK.219 and HEK293FT cells was performed with Lipofectamine RNAiMax reagent (Invitrogen) according to the manufacturer’s protocol. The 3×Flag-vIRF-1 and 3×Flag-vIRF-4 expression plasmids (56) were a generous gift from Zsolt Toth (University of Florida).

### Quantification of viral replication.

siRNAs targeting the cellular genes were transfected in iSLK.219 cells for 48 h, followed by KSHV reactivation by doxycycline (1 µg/ml) and sodium butyrate (NaB; 3 mM). After 24 h, the RFP fluorescence intensity was quantified using ImageJ software. The RFP signal intensity was normalized relative to that of nontargeting siRNA (siC).

### Construction of vIRF-1 and vIRF-4 mini-Turbo KSHV BAC16.

Recombinant KSHV was prepared by following a protocol for en passant mutagenesis with a two-step markerless red recombination technique (42). Briefly, a codon-optimized mini-TurboID coding sequence (Table 1) which also encodes 3× Flag tag was first cloned into a pBS SK vector (Thermo Fisher, Waltham, MA). The pEPkan-S plasmid was used as a source of the kanamycin cassette, which includes the I-SceI restriction enzyme site at the 5′ end of the kanamycin coding region (42). The kanamycin cassette was amplified with primer pairs listed in Table 1 and cloned into the mini-TurboID coding region at a unique restriction enzyme site. The resulting plasmid was used as a template for another round of PCR to prepare a transfer DNA fragment for markerless recombination with BAC16 (57). Recombinant BAC clones with insertion and also deletion of the kanamycin cassette in the BAC16 genome were confirmed by colony PCR with appropriate primer pairs. Recombination junctions and adjacent genomic regions were amplified by PCR, and the resulting PCR products were directly sequenced with the same primers to confirm in-frame insertion of the mini-TurboID cassette into the BAC DNA. The resulting recombinant BAC was confirmed by restriction enzyme digestions (HindIII and BglII), to determine if there were any large DNA deletions. Two independent BAC clones were generated for each mini-TurboID-tagged recombinant virus as biological replicates; one of the clones was used for protein identification. BAC DNAs were subsequently sequenced in their entirety.

**TABLE 1:** Primers, plasmid, and gene block DNA sequences used for BAC16 recombination^*a*^

Template and primer, probe, or fragment	Sequence (5′→3′)
Template: pEPkan-S Addgene (plasmid 41017)	
Mini-TurboID-Kan SalI-S	GCCCGTCGTCGACTCCACCAATCAGTACCTCTTGGATCGGATTGGGGAGTTGAAGAGCGGTAGGGATAACAGGGTAATCGATTT
Kan SalI-AS	AAAGTCGACGCCAGTGTTACAACCAATTAACC
3×Flag mini-TurboID codon-optimized fragment (KpnI/SacII) cloned into pBS SK+ vector with Gibson assembly	**ATG**GATTATAAGGATGATGACAAGGGGGACTATAAAGACGACGATAAAGGCGACTATAAGGACGATGATAAAGCGTCCATACCGCTGCTGAATGCAAAACAGATCCTGGGGCAGTTGGATGGTGGAAGCGTCGCAGTGCTGCCCGTCGTCGACTCCACCAATCAGTACCTCTTGGATCGGATTGGGGAGTTGAAGAGCGGTGATGCGTGCATCGCGGAGTACCAGCAAGCAGGCAGAGGTAGCCGCGGACGAAAATGGTTTAGTCCTTTTGGTGCGAACCTGTACCTCAGCATGTTCTGGAGGCTCAAGAGAGGCCCCGCGGCGATTGGACTTGGCCCAGTAATCGGGATCGTCATGGCTGAGGCGCTCAGAAAACTCGGAGCTGATAAGGTTAGAGTAAAATGGCCGAACGACCTTTATTTGCAAGACCGAAAATTGGCTGGGATATTGGTGGAACTTGCGGGCATTACCGGCGACGCGGCACAAATCGTCATAGGTGCCGGTATTAATGTGGCAATGCGCCGCGTTGAAGAGAGCGTGGTAAATCAGGGATGGATAACCCTGCAAGAGGCAGGAATCAACCTGGACCGCAACACCCTGGCTGCTATGCTCATTCGGGAACTGAGAGCTGCGTTGGAGCTCTTTGAACAGGAAGGGCTTGCACCGTACCTCAGTCGATGGGAAAAATTGGATAACTTCATAAATCGGCCTGTGAAACTCATCATAGGCGACAAGGAAATCTTTGGCATTAGTCGAGGGATTGATAAGCAAGGCGCACTCTTGCTCGAACAGGACGGAGTTATCAAACCTTGGATGGGTGGCGAAATTAGTCTCAGAAGTGCAGAGAAGGAGTTTAGCCGAGCGGAC***TAA***
Template: pBS-mini-TurboID Kan	
K9 mini-TurboID-S (vIRF-1)	*CTGTCGCCTCTCTATATCTGATGGCCGGTGGCTCCCCGGCATAGCTGTGCTTACCACTGGACATTGCGGCGCGAGCTAGTCTGGTTGCGGGACA***ATGGATTATAAGGATGATGACAAGGGGGAC**
K9 mini-TurboID-AS	*GTTCCCGGTGACCCTTGTGACAAACAAGGTTTTTTGGGTATCGCCCCAGGCGCCCCAAAAGGGTTCGGTCTTTGGCCTGGGTCCAT***GTCCGCTCGGCTAAACTCCTTCTCTG**
K10 mini-TurboID-S (vIRF-4)	*TAGCAAGAAGGGGGGCACTATAAGGCTCAGTCGGGACTGTGCCTCAAAGACGAACGCCGATCGGTTTCTGTGTCGGACC***ATGGATTATAAGGATGATGACAAGGGGGAC**
K10 mini-TurboID-AS	*AAACCAGGAAAAATAGGGAAACTTATTGTTTTCAAGGGCATCAATAATCCATAACGTGGCCCATTCTGAGCCACCGGCTTTAGG***GTCCGCTCGGCTAAACTCCTTCTCTG**

a Restriction enzyme sites used to clone the kanamycin cassette are underlined. Start and stop codons for the mini-TurboID coding sequence are marked with bold and underline. Italic letters indicate homology arms with the KSHV genome for recombination. Bold bases anneal to the cloned mini-TurboID cassette for amplification of the DNA fragment for recombination.

### Western blotting.

Cells were lysed in IP lysis buffer (25 mM Tris-HCl [pH 7.4], 150 mM NaCl, 1% NP-40, 1 mM EDTA, 5% glycerol) containing protease inhibitors (Roche, Basel, Switzerland). Total cell lysates (25 µg) were boiled in SDS-PAGE loading buffer, subjected to SDS-PAGE, and subsequently transferred to a polyvinylidene fluoride membrane (Millipore-Sigma, St. Louis, MO) using a semidry transfer apparatus (Bio-Rad, Hercules, CA). Streptavidin-HRP conjugate was used at a 1:3,000 dilution. The final dilutions or concentrations of the primary antibodies were 1:5,000 for anti-K-Rta rabbit serum, 1 µg/ml for anti-K8α (Santa Cruz, Santa Cruz, CA), 1 µg/ml for anti-ORF57 mouse monoclonal antibody (Santa Cruz), 1 µg/ml of anti-K8.1 mouse monoclonal (Santa Cruz), and 1:5,000 for anti-β-actin mouse monoclonal antibody (Millipore-Sigma, St. Louis, MO). Membrane washing and secondary antibody incubations were performed as described previously (58).

### Quantification of viral copy number.

Two hundred microliters of cell culture supernatant was treated with 12 µg/ml of DNase I for 15 min at room temperature to degrade nonencapsidated DNA. This reaction was stopped by the addition of EDTA to 5 mM, followed by heating at 70°C for 15 min. Viral genomic DNA was purified using a QIAamp DNA minikit according to the manufacturer’s protocol. Viral DNA was eluted in 100 µl of buffer AE. Four microliters of eluate was used for real-time qPCR to determine viral copy number, as described previously (58).

### Preparation of purified KSHV and virus infection.

iSLK cells latently infected with mini-TurboID-KSHVs were seeded in 8 to 10 15-cm dishes, stimulated with 1 µg/ml of doxycycline and 3 mM sodium butyrate for 24 h, and further incubated with culture media without stimuli for 72 h. The culture supernatant was centrifuged using a Beckman SW28 rotor (25,000 rpm for 2 h) with a 25% sucrose cushion. Virus pellets were dissolved in DMEM and further purified by discontinuous sucrose gradient (25 to 60%) centrifugation using a Beckman SW40Ti rotor (21,000 rpm for 16 h). Virus pellets were dissolved in DMEM for infection studies. HEK293FT cells were infected with 1 genomic copy of virus per cell in DMEM. Twenty-four hours postinfection, cells were washed with PBS and incubated for 24 h in complete media. After 48 h postinfection, cells were trypsinized and washed twice with PBS. Cells were resuspended in PBS containing 1% bovine serum albumin (BSA) and 1 mM EDTA, and recombinant viral infection was analyzed with flow cytometry (BD Accuri) and FlowJo software.

### Real-time RT-PCR.

Total RNA was isolated using a Quick-RNA miniprep kit (Zymo Research, Irvine, CA). First-strand cDNA was synthesized using high-capacity cDNA reverse transcription (RT) kit (Thermo Fisher, Waltham, MA). Gene expression was analyzed by real-time qPCR using specific primers for KSHV open reading frames (ORFs) designed by Fakhari and Dittmer (59). We used 18S rRNA as an internal standard to normalize viral gene expression.

### Affinity purification of biotinylated proteins.

Affinity purification was done with streptavidin-coated magnetic beads (Thermo Fisher). Briefly, 150 µl of magnetic beads/sample were prewashed with RIPA lysis buffer (150 mM NaCl, 5 mM EDTA [pH 8], 50 mM Tris [pH 8], 1% NP-40, 0.5% sodium deoxycholate, 0.1% SDS) 3 times. A total of 3 mg of whole-cell lysate was incubated with prewashed streptavidin beads at room temperature for 1 h for rotation. The beads were collected using a magnetic stand and washed three times with wash buffer according to the manufacturer’s protocol. Finally, beads were resuspended in 200 µl of wash buffer and sent to the UC Davis proteomics core for on bead digestion and liquid chromatography-tandem mass spectrometry (LC-MS/MS) analysis.

### MS sample preparation.

Protein samples on magnetic beads were washed four times with 200 µl of 50 mM ammonium bicarbonate (AMBIC) with a 20-min shake time at 4°C in between each wash. Roughly 2.5 µg of trypsin was added to the beads and AMBIC, and the samples were digested overnight at a shake speed of 800 rpm. After overnight digestion, the supernatant was removed, and the beads were washed once with enough 50 mM ammonium bicarbonate to cover. After 20 min with gentle shaking, the wash was removed and combined with the initial supernatant. The peptide extracts were reduced in volume by vacuum centrifugation and a small portion of the extract was used for fluorometric peptide quantification (Thermo Scientific Pierce). One microgram of sample based on the fluorometric peptide assay was loaded for each LC-MS analysis.

Digested peptides were analyzed by LC-MS/MS on a Thermo Scientific Q Exactive Orbitrap mass spectrometer in conjunction with a Proxeon Easy-nLC II high-performance liquid chromatograph (HPLC; Thermo Scientific) and Proxeon nanospray source. The digested peptides were loaded onto a 100-μm by 25-mm Magic C_18_ 100-Å 5U reverse-phase trap, where they were desalted online before being separated using a 75-μm by 150-mm Magic C_18_ 200-Å 3U reverse-phase column. Peptides were eluted using a 60-min gradient with a flow rate of 300 nl/min. An MS survey scan was obtained for the *m/z* range 300 to 1,600, and MS/MS spectra were acquired using a top-15 method, where the top 15 ions in the MS spectra were subjected to high-energy collisional dissociation (HCD). An isolation mass window of 2.0 *m/z* was used for precursor ion selection, and a normalized collision energy of 27% was used for fragmentation. A 15-s duration was used for dynamic exclusion.

### MS/MS analysis.

Tandem mass spectra were extracted and charge state deconvoluted by Proteome Discoverer (Thermo Scientific). All MS/MS samples were analyzed using X!. All MS/MS samples were analyzed using X! Tandem (The Global Proteome Machine [https://thegpm.org]; version X! Tandem Alanine [2017.2.1.4]). X! Tandem was set up to search the human and Kaposi’s sarcoma herpesvirus database (149,182 entries) assuming the digestion enzyme trypsin. X! Tandem was searched with a fragment ion mass tolerance of 20 ppm and a parent ion tolerance of 20 ppm. Carbamidomethyl of cysteine and selenocysteine was specified in X! Tandem as a fixed modification. Glu→pyro-Glu of the N terminus, ammonia loss of the N terminus, Gln→pyro-Glu of the N terminus, deamidation of asparagine and glutamine, oxidation of methionine and tryptophan, and dioxidation of methionine and tryptophan were specified in X! Tandem as variable modifications.

Scaffold (version Scaffold_4.8.4; Proteome Software Inc., Portland, OR) was used to validate MS/MS-based peptide and protein identifications. Peptide identifications were accepted if they could be established at more than 98.0% probability by the Scaffold Local FDR algorithm. Peptide identifications were also required to exceed specific database search engine thresholds. Protein identifications were accepted if they could be established at more than 5.0% probability to achieve a false-discovery rate (FDR) less than 5.0% and contained at least 2 identified peptides. Protein probabilities were assigned by the Protein Prophet algorithm (60). Proteins that contained similar peptides and could not be differentiated based on MS/MS analysis alone were grouped to satisfy the principles of parsimony. Proteins sharing significant peptide evidence were grouped into clusters.

Scaffold software was used for normalization of the peptide with the following steps. (i) Calculate the total number of spectra in each BioSample. (ii) Calculate the average number of spectra across all BioSamples. (iii) Multiply each spectrum count in each sample by the average count over the BioSample’s total spectrum count.

### Pathway analysis.

The proteins identified as interacting with vIRF-1 and vIRF-4 were used for gene ontology. The top gene ontology processes were enriched by the Metascape web-based platform, and Metascape software was used for gene ontology analysis (61).

### Statistical analysis.

Results are shown as means ± standard deviations (SD) from at least three independent experiments. Data were analyzed using unpaired Student’s *t* test or using analysis of variance (ANOVA) followed by Tukey’s honestly significant difference (HSD) test. A *P* value of <0.05 was considered statistically significant.

## Supplementary Material

Supplemental file 1

Supplemental file 2

Supplemental file 3
