# Supplementary material for: Proximity Biotin Labeling Reveals Kaposi’s Sarcoma-Associated Herpesvirus Interferon Regulatory Factor Networks
Source: J Virol. 2021 Apr 12;95(9):e02049-20. doi: 10.1128/JVI.02049-20 (PMC8104114; doi:10.1128/JVI.02049-20)
Supplement: Supplemental file 3 [file JVI.02049-20-s0001.pdf]

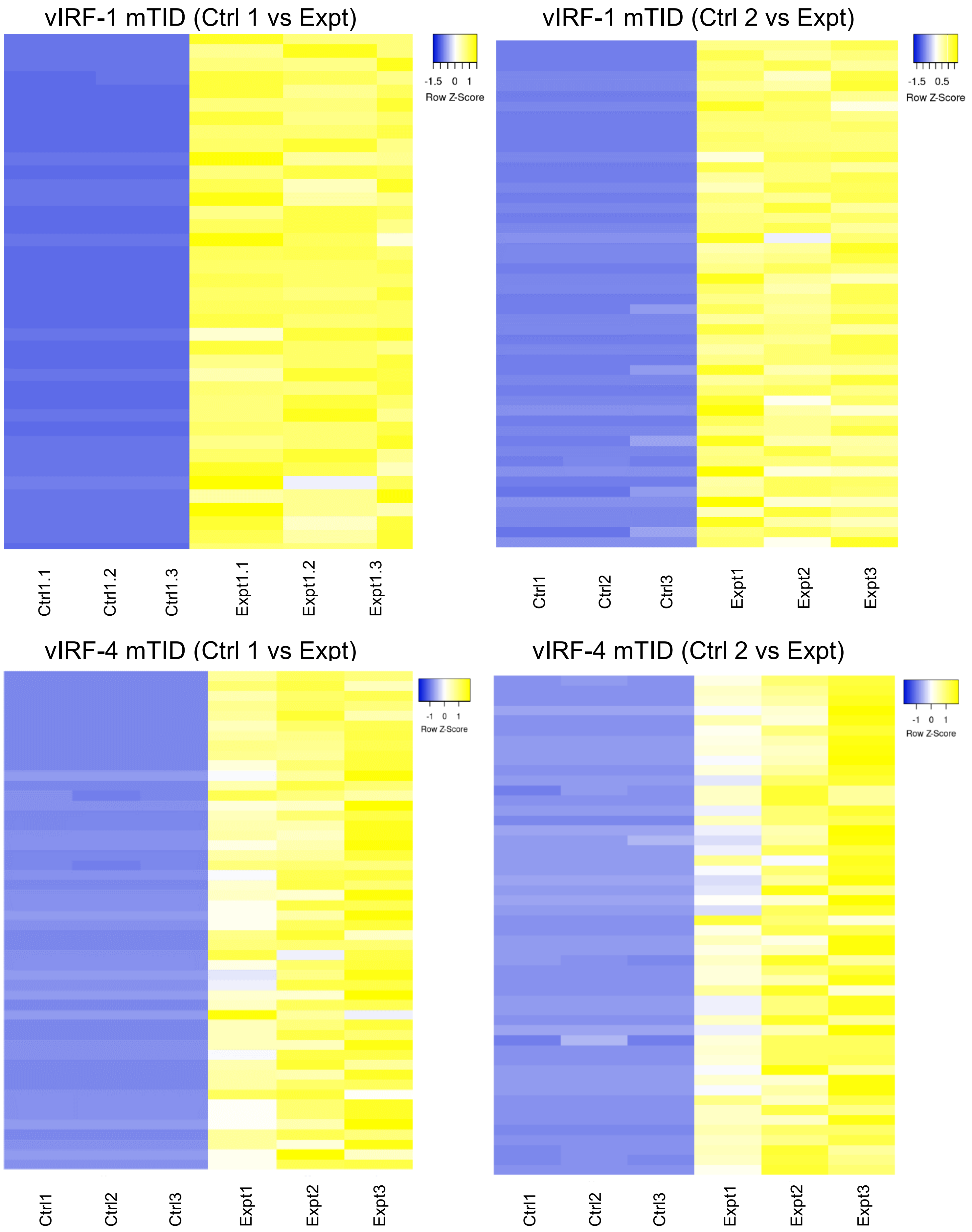

Supplementary Figure 1: Heatmap was generated for vIRF-1 (upper panel) and vIRF-4 (lower panel) using peptide count for Ctrl1, Expt and Ctrl2 samples.

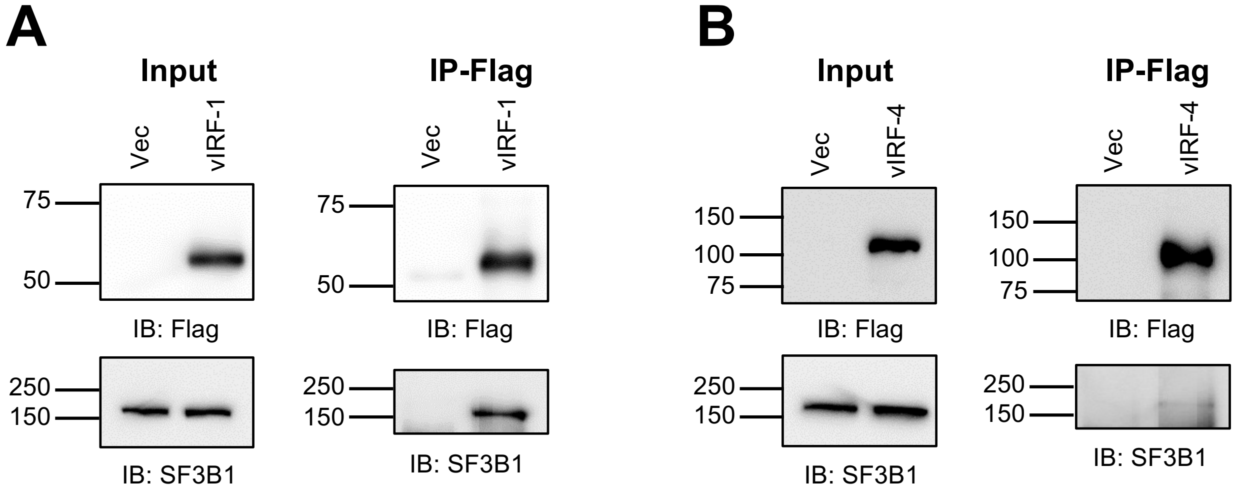

Supplementary Figure 2: **Co-immunoprecipitation between SF3B1 and vIRF.** HEK293T cells were transfected with flag tagged vIRF-1 (**A**) and v-IRF-4 (**B**) for 24 h. v-IRF1 and v-IRF4 was immunoprecipitated by flag-agarose beads. Precipitated proteins were probed with either anti-Flag antibody or anti-SF3B1 antibody..
